# Cerebrospinal Fluid Concentration of Key Autophagy Protein Lamp2 Changes Little During Normal Aging

**DOI:** 10.3389/fnagi.2018.00130

**Published:** 2018-05-08

**Authors:** David A. Loeffler, Andrea C. Klaver, Mary P. Coffey, Jan O. Aasly

**Affiliations:** ^1^Department of Neurology, Beaumont Hospital-Royal Oak, Beaumont Health, Royal Oak, MI, United States; ^2^Department of Biostatistics, Beaumont Hospital-Royal Oak, Beaumont Health, Royal Oak, MI, United States; ^3^Department of Neurology, St. Olav’s Hospital, Trondheim, Norway

**Keywords:** aging, autophagy, CSF, HSPA8, lamp2, oxidative stress

## Abstract

Autophagy removes both functional and damaged intracellular macromolecules from cells via lysosomal degradation. Three autophagic mechanisms, namely macroautophagy, chaperone-mediated autophagy (CMA), and microautophagy, have been described in mammals. Studies in experimental systems have found macroautophagy and CMA to decrease with normal aging, despite the fact that oxidative stress, which can activate both processes, increases with normal aging. Whether autophagic mechanisms decrease in the human brain during normal aging is unclear. The primary objective of this study was to examine the association of a major autophagy protein, lysosome-associated membrane glycoprotein (lamp2), with age in cerebrospinal fluid (CSF) samples from healthy subjects. Lamp2 consists of three isoforms, lamp2a, 2b and 2c, all of which participate in autophagy. Lamp2’s CSF concentration decreases in Parkinson’s disease (PD) and increases in Alzheimer’s disease (AD), but whether its CSF concentration changes during normal aging has not been investigated. Our secondary objectives were to examine the associations of lamp2’s CSF concentration with CSF levels of the molecular chaperone heat shock 70-kDa protein (HSPA8), which interacts with lamp2a in CMA, and oxidative stress markers 8-hydroxy-2′-deoxyguanosine (8-OHdG), 8-isoprostane (8-ISO) and Total Antioxidant Capacity (TAC) in healthy subjects. We found lamp2’s observed associations with these variables to be weak, with all Kendall’s tau-b absolute values ≤0.20. These results suggest that CSF lamp2 concentration changes little during normal aging and does not appear to be associated with HSPA8 or oxidative stress. Further studies are indicated to determine the relationship between CSF lamp2 concentration and brain autophagic processes.

## Introduction

Proteostasis involves regulation of protein transcription, translation, folding, trafficking, processing, assembly/disassembly, localization, and degradation (Douglas and Dillin, 2010). Cells attempt to maintain proteostasis via the autophagy-lysosomal pathway and the ubiquitin-proteasome system (Xilouri and Stefanis, 2016). Three autophagic processes, namely macroautophagy, chaperone-mediated autophagy (CMA) and microautophagy have been described in mammals (Cuervo, 2004). Based on studies in rat livers and human fibroblast cultures, macroautophagy and CMA are thought to decrease with normal aging (Cuervo and Dice, 2000; Del Roso et al., 2003). If these processes decrease in the brain during normal aging, this could contribute to aging being a primary risk factor for the two most prevalent neurodegenerative disorders, Alzheimer’s disease (AD) and Parkinson’s disease (PD; Jeppesen et al., 2011; Xilouri and Stefanis, 2016). The few investigations of age-related changes in brain autophagy have produced conflicting results. Genome-wide analyses of human brain specimens found downregulation of a key macroautophagy protein, beclin-1 (Shibata et al., 2006), and of major autophagy genes including Atg5 and Atg7 (Lipinski et al., 2010b); the latter study also found upregulation of genes involved in regulation and mediation of the mitogen-activated protein (MAP) kinase pathway, which was predicted in an earlier study (Lipinski et al., 2010a) to result in suppression of autophagy. Conversely, a proteomics study of human cerebrospinal fluid (CSF) found that three proteins with the gene ontology classification of “autophagy” namely myoglobin, MMP8 and HMW kininogen (none of which is a major autophagy protein), increased with age (Baird et al., 2012). An age-related increase in macroautophagy in the rat brain has also been reported (Gamerdinger et al., 2009).

There are no established biomarkers in cerebrospinal fluid (CSF) for monitoring brain autophagy. Beclin-1, p62 and LC3-II have been suggested as macroautophagy biomarkers (Karim et al., 2007; Pattingre et al., 2008; Li et al., 2015; Au et al., 2017) but, to our knowledge, age-related changes in these proteins have not been examined in CSF. We recently reported (Loeffler et al., 2016) that the CSF concentration of heat shock 70-kDa protein (HSPA8, also known as hsc70 and hsc73), a molecular chaperone involved in CMA, decreases with aging, but whether changes in CSF HSPA8 levels reflect changes in brain CMA is unknown. In the present study we explored changes in the CSF concentration of another autophagy protein, lysosome-associated membrane glycoprotein 2 (lamp2), during normal aging. Lamp2 has three isoforms, lamp2a, 2b and 2c, which are generated by alternative splicing of the *LAMP2* gene and differ in their C-terminus sequences (Gough et al., 1995). All three isoforms participate in autophagy; lamp2a’s binding by the substrate protein—heat shock 70-kDa protein (HSPA8) complex is rate-limiting for CMA (Cuervo and Dice, 1996), lamp2b is involved in macroautophagy (and may be required for autophagosome-lysosome fusion; Nishino et al., 2000; Rowland et al., 2016), and lamp2c is a receptor for autophagic degradation of DNA and RNA (Fujiwara et al., 2015). Lamp2’s CSF concentration has been reported to decrease in PD (Boman et al., 2016; Klaver et al., 2018) and to increase in AD (Armstrong et al., 2014), in accordance with reports of decreased lamp2a and HSPA8 (referred to in that study as hsc70) in PD brain specimens (Alvarez-Erviti et al., 2010) and increased transcription of positive regulatory genes for autophagy in AD brain specimens (Lipinski et al., 2010b). Lamp2’s gene expression in human leukocytes decreases with normal aging (Huang et al., 2012), but whether its CSF concentration changes during normal aging is unknown. Our primary objective in this study was to examine this issue. Our secondary objectives were to examine lamp2’s correlations, in CSF from healthy subjects, with HSPA8 and with markers of oxidative stress (8-hydroxy-2′-deoxyguanosine [8-OHdG], 8-isoprostane [8-ISO], and Total Antioxidant Capacity [TAC]). TAC includes nonenzymatic low molecular weight antioxidants such as ascorbic acid and glutathione (Alho et al., 1998; Bartosz, 2003); because a decrease in TAC could result in elevated oxidative stress, it may be an indirect marker for oxidative stress (Mandrioli et al., 2006). Oxidative stress increases in the aged brain (Navarro et al., 2002; Vanguilder and Freeman, 2011) and can activate both CMA and macroautophagy (Kiffin et al., 2004; Kaushik et al., 2010). In a previous study with these samples (Loeffler et al., 2016), we found the CSF concentration of the oxidative stress marker 8-OHdG to increase with age (Spearman rho = 0.61).

## Materials and Methods

### Study Subjects

Details of the subjects whose CSF samples were used in this study were reported previously (Loeffler et al., 2016). The subjects were recruited by neurologist Jan Aasly, at St. Olav’s Hospital, Trondheim, Norway. They were tested for and lacked known PD-related mutations in the *LRRK2*, *PARK2*, *PARK7*, *PINK1* and *SNCA* genes, and had no detectable cognitive or neurological impairments. All procedures involving the study subjects, including obtaining of written informed consent, were performed at St. Olav’s Hospital in accordance with the Declaration of Helsinki of 1975 and its subsequent amendments. The study was approved by the Regional Committee for Medical Research Ethics, Central Norway, for the procedures performed at St. Olav’s Hospital. Lumbar CSF samples were obtained using Parkinson’s Progression Markers Initiative (PPMI) biospecimen collection procedures (Parkinson’s Progression Markers Initiative, 2014). The study was given exempt status from the Institutional Review Board of Beaumont Health (Royal Oak, MI, USA) for the procedures performed in the Neurology Research Laboratory at Beaumont Hospital-Royal Oak (MI, USA), where lamp2 was measured in de-identified CSF samples.

### Lamp2 Measurements

Lamp2 was measured using the ELISA Kit for Lysosomal Associated Membrane Protein 2 (LAMP2; cat. # SEB464Hu) from Cloud-Clone Corp. (Katy, TX, USA). The lower limit of detection for lamp2 in the ELISA kit was stated by the manufacturer to be 26.2 pg/mL. The standard curve in the assay ranged from 62.5 pg/mL to 4000 pg/mL. The lamp2 concentration of each sample was measured in duplicate after diluting the sample with an equal volume of 0.01 M PBS buffer, pH 7.2; the lamp2 concentrations used in statistical analyses were the means of these duplicate measurements. The standard curve was generated using Softmax Pro software (version 3.0; Molecular Devices Corp., Sunnyvale, CA, USA) using the log-log function as recommended by the manufacturer, and the concentration of lamp2 in each sample was calculated by Softmax. One sample produced a mean optical density value which was below the value for the lowest point on the standard curve; for statistical purposes, the sample was assigned a concentration of 26.2 pg/mL, the lower limit of sensitivity for the assay. After accounting for the dilution factor, the concentration of the sample was listed as 52.4 pg/mL.

### Statistics

Measures were summarized with means ± SDs for variables which were normally distributed, and with medians and ranges for variables which were not normally distributed. Lamp2 values were distributed abnormally (strongly skewed right with extreme outliers); natural-log transformation produced a reasonably normal distribution. Kendall’s rank-correlation coefficient (Kendall’s tau-b) with associated 95% confidence intervals (CI), and scatterplots with best-fit regression lines and locally weighted scatterplot smoother (LOWESS) curves were used to examine lamp2’s associations with age, HSPA8, 8-OHdG, 8-ISO and TAC. Differences for lamp2 concentrations between male and female subjects were examined with *t*-tests. Original (non-transformed) lamp2 values were used for Kendall’s tau-b calculations, while log-transformed lamp2 values were used for scatterplots and gender comparisons. Statistical analysis used The SAS System for Windows version 9.3 (SAS Institute Inc., Cary, NC, USA) and Minitab Release 14 (Minitab Inc., State College, PA, USA) was used for graphs.

## Results

### Associations of CSF Lamp2 Concentration With Other Variables

Numeric summaries for age and lamp2, HSPA8, 8-OHdG, 8-ISO, and TAC concentrations are shown in Table 1, and lamp2’s associations with the other variables are shown in Table 2. Lamp2 was poorly correlated with age (Kendall’s tau-*b* = 0.16; 95% CI for tau-b = [−0.08, 0.40]) and with the other variables (all Kendall’s tau-b absolute values ≤ 0.20). The 95% CIs for lamp2’s associations with age [−0.08, 0.40] and with 8-OHdG [−0.01, 0.41] do not rule out the possibility of moderate positive associations.

**Table 1:** Age and cerebrospinal fluid (CSF) lysosome-associated membrane glycoprotein 2 (lamp2), heat shock 70-kDa protein (HSPA8), 8-OHdG, 8-ISO and TAC concentrations in study subjects.

Variable	Summary measure
Age (years)—median, range	55.5 (20–75)
lamp2 (pg/mL)—median, range	350.8 (52.4–10,561)
HSPA8 (ng/mL)—mean ± SD	0.5 ± 0.2
8-OHdG (pg/mL)—mean ± SD	811.3 ± 160.8
8-ISO (pg/mL)—median, range	6.5 (3.2–16.7)
TAC (mM)—median, range	0.4 (0.2–0.6)

*Study subjects were 18 males and 16 females. The data for HSPA8, 8-OHdG, 8-ISO and TAC were taken from our previous study with these samples (Loeffler et al., 2016). 8-OHdG, 8-hydroxy-2′-deoxyguanosine; 8-ISO, 8-isoprostane; TAC, Total Antioxidant Capacity*.

**Table 2:** Kendall’s rank-correlation coefficients of CSF lamp2 concentrations with age, HSPA8 and oxidative stress measures in study subjects.

Variable	Kendall’s Tau-b	95% CI for Kendall’s Tau-b
Age	0.16	(−0.08, 0.40)
HSPA8	−0.15	(−0.39, 0.09)
8-OHdG	0.20	(−0.01, 0.41)
8-ISO	−0.13	(−0.33, 0.07)
TAC	0.09	(−0.17, 0.34)

*CSF lamp2 concentration was weakly correlated with the other variables. HSPA8, heat shock 70-kDa protein 8; 8-OHdG, 8-hydroxy-2′-deoxyguanosine; 8-ISO, 8-isoprostane; TAC, Total Antioxidant Capacity*.

**Figure 1:**
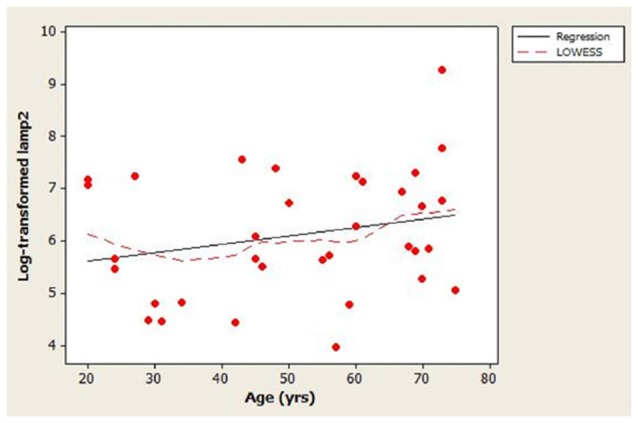
Distribution of cerebrospinal fluid (CSF) log-transformed lamp2 concentrations as a function of age in study subjects. A best-fit regression line and locally weighted scatterplot smoother (LOWESS) curve are shown; the LOWESS curve closely followed the regression line.

**Figure 2:**
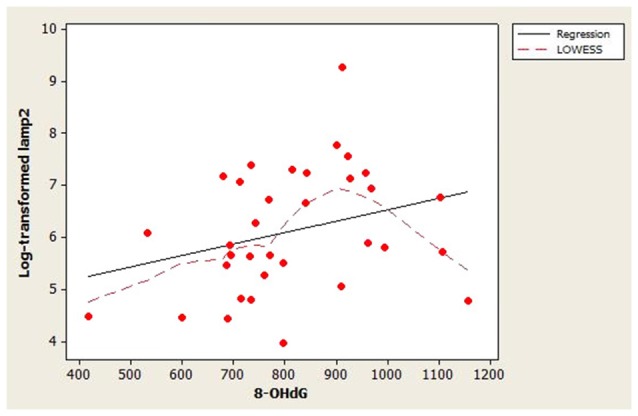
Distribution of CSF log-transformed lamp2 concentrations as a function of 8-hydroxy-2′-deoxyguanosine (8-OHdG) concentrations in study subjects. A best-fit regression line and LOWESS curve are shown. The LOWESS curve suggested that log-transformed lamp2 may increase with 8-OHdG to approximately 900 pg/mL; after that point it may level off or decrease.

The scatterplot of log-transformed lamp2 vs. age (Figure 1) indicated that the LOWESS curve closely followed the regression line, suggesting that the weak positive relationship that we observed between lamp2 and age was no more complex than linear. Conversely, in the scatterplot for log-transformed lamp2 vs. 8-OHdG (Figure 2), the LOWESS curve was steeper than the regression line to an 8-OHdG concentration of approximately 900 pg/mL, suggesting that lamp2 might increase with 8-OHdG to this 8-OHdG concentration, then level off or decrease. Scatterplots of log-transformed lamp2 vs. 8-ISO, TAC, and HSPA8 (not shown) did not suggest an association of lamp2 with any of these variables.

### Gender Differences in CSF Lamp2 Concentration

No significant differences were found for CSF lamp2 concentration between genders (*p* = 0.80). Because the male study subjects tended to be younger than the female study subjects, the gender comparison was limited to subjects ≥40 years old, namely 16 females (mean age 60.3) and nine males (mean age 60.1).

## Discussion

The main finding in this study was that the association between CSF lamp2 concentration and age appeared to be weak in healthy subjects. The scatter plot for the distribution of log-transformed lamp2 vs. age (Figure 1) indicated large variability in lamp2 concentrations among similar-aged individuals. Age could explain, at best, only a small proportion of this variability (adjusted *R*^2^ = 1.4%). Whether the lack of age-related changes in CSF lamp2 reflects a similar lack of change in brain lamp2 and/or brain autophagic processes during normal aging is unknown; such a determination would require measurement of lamp2 on lysosomal membranes, as well as assessment of CMA and macroautophagy activities, in normal brain specimens across a wide age range.

Because the concentration of lamp2 is the sum of the concentrations of all three lamp2 isoforms, the lack of change that we found in CSF lamp2 concentration during normal aging does not necessarily indicate a lack of change in the concentrations of its isoforms. A study measuring mRNA for lamp2’s isoforms in normal human anterior cingulate and occipital cortex (Murphy et al., 2015) found that only 5% of lamp2 mRNA encoded for lamp2a, whereas 85% encoded for lamp2c. Whether lamp2’s isoforms are similarly distributed in CSF is unknown. If lamp2a accounts for only 5% of lamp2’s CSF concentration, then it may be difficult to detect by standard techniques such as ELISA or western blot. No commercial ELISAs are available for measuring lamp2’s isoforms.

The correlation between lamp2 and HSPA8 was −0.15. It is unclear if a stronger association between these proteins should have been expected. Activation of CMA is often mediated by increases in the lysosomal levels of both lamp2a and HSPA8 (Agarraberes et al., 1997), but as discussed above, changes in lamp2’s CSF concentration may not reflect those of lamp2a. HSPA8 is the main housekeeping member of the heat shock protein 70 family and is involved in many other processes in addition to CMA (Stricher et al., 2013), so its concentration in CSF may not reflect brain CMA activity.

Lamp2’s correlations with the oxidative stress markers were also weak. This suggests that lamp2 CSF concentration in healthy individuals may not be associated with, and may not be influenced by, oxidative stress. Although the LOWESS curve in Figure 2 suggested that lamp2 might increase with 8-OHdG to approximately 900 pg/mL 8-OHdG, this finding requires confirmation.

The number of subjects in this study was sufficient for investigating lamp2’s associations with age, HSPA8, and oxidative stress markers. Although the CI for lamp2’s associations with age and 8-OHdG do not rule out the possibility of moderate positive associations, they suggest that negative associations between these variables are unlikely. This was a cross-sectional study which measured CSF lamp2 at a single age in each subject; a study measuring each subject’s CSF lamp2 at multiple time points (e.g., over multiple decades) might have increased our ability to detect age-related changes in lamp2.

Recent studies have indicated a relationship between autophagic processes and lipid metabolism. In experimental models, lipid loading exerts inhibitory effects on both macroautophagy and CMA in hepatocytes (Koga et al., 2010; Rodriguez-Navarro et al., 2012). In the latter study, the inhibitory effects of lipid challenge on CMA were ascribed to decreased stability of lamp2a at lysosomal membranes, which resulted in lowering of its concentration there. Whether increased lipid intake similarly causes changes in the lamp2a and/or total lamp2 concentrations in the CNS is unknown. All of our subjects had similar basal metabolic index classifications, and we were not aware of differences in their eating habits. However, because the basal metabolic rate decreases each decade after age 20 by 1%–2% (Manini, 2010), we cannot rule out the possibility that this could have influenced the correlation (which was weak) that we found between CSF lamp2 and subject age.

We conclude that CSF lamp2 concentration appears to change little during normal aging, and it appears to be poorly associated with CSF concentrations of HSPA8 and of oxidative stress markers. Further studies are indicated to clarify the effects of aging on autophagic processes in the human brain and to identify CSF biomarkers for these processes.

## Author Contributions

DAL directed the study and prepared the original and revised manuscript. ACK performed the assays, collated the data and reviewed the manuscript. MPC performed the statistical analysis and assisted with manuscript revisions. JOA recruited the patients, collected the CSF samples and reviewed the manuscript.

## Conflict of Interest Statement

The authors declare that the research was conducted in the absence of any commercial or financial relationships that could be construed as a potential conflict of interest.
